# Lipidomics Reveals Cisplatin-Induced Renal Lipid Alterations during Acute Kidney Injury and Their Attenuation by Cilastatin

**DOI:** 10.3390/ijms222212521

**Published:** 2021-11-20

**Authors:** Estefanía Moreno-Gordaliza, Maria Dolores Marazuela, Óscar Pastor, Alberto Lázaro, María Milagros Gómez-Gómez

**Affiliations:** 1 Department of Analytical Chemistry, Faculty of Chemistry, Universidad Complutense de Madrid, 28040 Madrid, Spain; doloresl@ucm.es (M.D.M.); mmgomez@ucm.es (M.M.G.-G.); 2 Servicio de Bioquímica Clínica, UCA-CCM, Instituto Ramón y Cajal de Investigación Sanitaria (IRYCIS), CIBER Fisiopatología de la Obesidad y Nutrición (CIBERobn), Hospital Universitario Ramón y Cajal, 28034 Madrid, Spain; oscar.pastor@salud.madrid.org; 3 Renal Physiopathology Laboratory, Department of Nephrology, Instituto de Investigación Sanitaria Gregorio Marañón, Hospital General Universitario Gregorio Marañón, 28007 Madrid, Spain; alberlaz@ucm.es; 4 Department of Physiology, School of Medicine, Universidad Complutense de Madrid, 28040 Madrid, Spain

**Keywords:** lipidomics, cisplatin, acute kidney injury, cilastatin, nephroprotection, biomarkers, cholesterol esters, sphingolipids, phospholipids, chemotherapy

## Abstract

Nephrotoxicity is a major complication of cisplatin-based chemotherapy, leading to acute kidney injury in ca. 30% of patients, with no preventive intervention or treatment available for clinical use. Cilastatin has proved to exert a nephroprotective effect for cisplatin therapies in in vitro and in vivo models, having recently entered clinical trials. A deeper understanding at the molecular level of cisplatin-induced renal damage and the effect of potential protective agents could be key to develop successful nephroprotective therapies and to establish new biomarkers of renal damage and nephroprotection. A targeted lipidomics approach, using LC-MS/MS, was employed for the quantification of 108 lipid species (comprising phospholipids, sphingolipids, and free and esterified cholesterol) in kidney cortex and medulla extracts from rats treated with cisplatin and/or cilastatin. Up to 56 and 63 lipid species were found to be altered in the cortex and medulla, respectively, after cisplatin treatment. Co-treatment with cilastatin attenuated many of these lipid changes, either totally or partially with respect to control levels. Multivariate analysis revealed that lipid species can be used to discriminate renal damage and nephroprotection, with cholesterol esters being the most discriminating species, along with sulfatides and phospholipids. Potential diagnostic biomarkers of cisplatin-induced renal damage and cilastatin nephroprotection were also found.

## 1. Introduction

Nephrotoxicity is a serious side effect of cisplatin chemotherapy [1], with acute kidney injury (AKI) being developed in ca. 30% of the treated patients [2]. This is also the main dose-limiting factor and an important drawback of cisplatin-based therapies, one of the most relevant treatments for solid tumors [2]. Cisplatin accumulates and causes injury to renal proximal tubule epithelial cells (RPTECs), and the damage particularly locates in segment S3 of the proximal tubule, descending from the renal cortex towards the corticomedullary junction and outer medulla [3]. However, direct damage to medullary Henle’s loop has also been suggested [4,5]. A series of cellular events take place in relation to renal tubule cell injury, following cisplatin uptake, including oxidative stress, nitrosative stress, vascular injury, inflammation, mitochondrial damage or inhibition of Na^+^, K^+^-ATPase in the cell membrane, and endoplasmic reticulum stress, followed by proximal tubule cell death mainly by apoptosis (both involving intrinsic and extrinsic pathways) or necrosis, leading to loss of renal function [2,6,7]. This is manifested in the decrease in the glomerular filtration rate (GFR) and serum magnesium and potassium levels, with increased blood urea nitrogen (BUN) and serum creatinine [2].

Various approaches have been investigated for nephroprotection during cisplatin therapies both in vitro and in vivo, based on a number of molecular targets [7,8,9]. However, the possible disturbance of the cytotoxic effect of cisplatin on tumor cells and an often incomplete protective effect have limited the development of renoprotectors [9]. As a result, there is no intervention for clinical use that successfully prevents or treats cisplatin-induced AKI [7]. A deeper understanding of the molecular pathways related to cisplatin-induced AKI and potential targets for nephroprotection seems crucial in this regard.

Cilastatin has been proven to effectively protect kidney from the damage caused by several agents, including cisplatin [10,11], gentamicin [12], vancomycin [13], cyclosporine A, and tacrolimus [14], in both in vitro and in vivo models. Cilastatin exerts its protective effect by selectively inhibiting renal dehydropeptidase I (DHP-I) located in the lipid rafts on the brush border of the RPTECs [11]. As a result, processes involving membrane externalization such as Fas-mediated extrinsic apoptosis are impaired by cilastatin, and progression of renal damage after a toxic insult is detained [11]. Aside from extrinsic apoptosis impairment by cilastatin, oxidative stress, inflammation, and nephrotoxic compounds’ accumulation were all found to be decreased [10,11,12,13,15,16]. Thus, renal function remains preserved under co-treatment with cisplatin and cilastatin [11]. Recently, cilastatin has successfully completed phase I clinical trials for its use as nephroprotector and is about to enter phase II trials. A promising recent study also revealed that cilastatin (administered as imipenem + cilastatin) may achieve a renoprotective effect in patients with peritoneal carcinomatosis during hyperthermic intraperitoneal cisplatin treatment [17].

In recent years, omics technologies, especially proteomics [18,19] and metabolomics [20,21,22,23], have proved useful for the discovery of novel early diagnostic biomarkers of AKI, aside from BUN or creatinine in serum [18,20]. In addition, metabolomics analysis can provide valuable information for the understanding of cisplatin mechanisms of nephrotoxicity [5,24] and the therapeutic effect of potential nephroprotectors [25,26,27].

In particular, lipidomics can also provide relevant information regarding kidney disease [28], considering that lipids present key structural, cell signaling, and metabolism roles, and they can be altered in cells, tissues, and biofluids under renal pathological conditions [29,30]. In this regard, increased levels of triglyceride and non-esterified fatty acids were found in the kidney during cisplatin treatment [31], in addition to total neutral lipids [32]. Elevated levels of sphingolipids—ceramides (Cer) and hexosylceramides (HexCer)—were also observed in the renal cortex during cisplatin-induced AKI [33]. On the other hand, phospholipid species, including phosphatidylcholine (PC), phosphatidylethanolamine (PE), and lysophosphatidylcholine (LPC) species, were also found to be altered in kidney tissue or cells during cisplatin treatment [26]. Levels of diverse LPC species were also found to be altered in rat serum after cisplatin administration [23]. Moreover, a mass spectrometry imaging non-targeted semi-quantitative approach allowed comparing the lipid distribution in rat kidney sections and suggested that cisplatin led to alterations on diverse phospholipids (PC, PE, phosphatidylglycerols (PG), phosphatidylinosytols (PI), phosphatidylserines (PS), phosphatidic acids (PA)), sulfatides (Sulf), or cardiolipins) [34] (showing different behavior in the cortex and medulla), while co-treatment with cilastatin tended to attenuate some of these changes [4].

In this work, we have gone a step further and taken a closer look at important structural lipids in the kidney, such as phospholipids (PC, LPC and PE), sphingolipids (Sulf, sphingomyelins (SM), dihydrosphingomyelins (dhSM), Cer, dihydroceramides (dhCer), HexCer, dihydrohexosylceramides (dhHexCer)), and free cholesterol (FC) and its esterified forms (cholesterol esters (CE)), covering new lipid classes and species and using a more reliable targeted quantitative approach based on liquid chromatography coupled to tandem mass spectrometry (LC-MS/MS) analysis. A total of 108 lipid species were quantified both on renal cortex and medulla extracts from treated rats, in order to better understand the nephrotoxic effect of cisplatin and the nephroprotective effect of cilastatin. New information on the AKI-associated cisplatin impact on renal lipid species was obtained and additional insights were gained into the protective effect of cilastatin, which led to an attenuation of some of the specific lipid alterations caused by cisplatin either in the renal cortex or medulla. Diverse potential biomarkers of cisplatin-induced renal damage and nephroprotection with cilastatin were also found.

## 2. Results

### 2.1. Cilastatin Protective Effect against Cisplatin-Induced Renal Damage

Assessment of renal function in cisplatin and/or cilastatin treated rats was first carried out employing several biochemical indicators in serum and urine. As shown in Table 1, serum creatinine, BUN, urine proteins, urine volume, and fractional excretion of sodium and potassium were significantly increased owing to cisplatin treatment compared with the control group, while co-treatment with cilastatin and cisplatin led to their decrease compared with control levels. On the other hand, GFR was decreased in cisplatin-treated rats with respect to control samples, whereas co-administration of cilastatin diminished this effect. These results confirm renal damage induced by cisplatin and protection by cilastatin. Cilastatin alone did not show any effect on renal function parameters.

**Figure 1:**
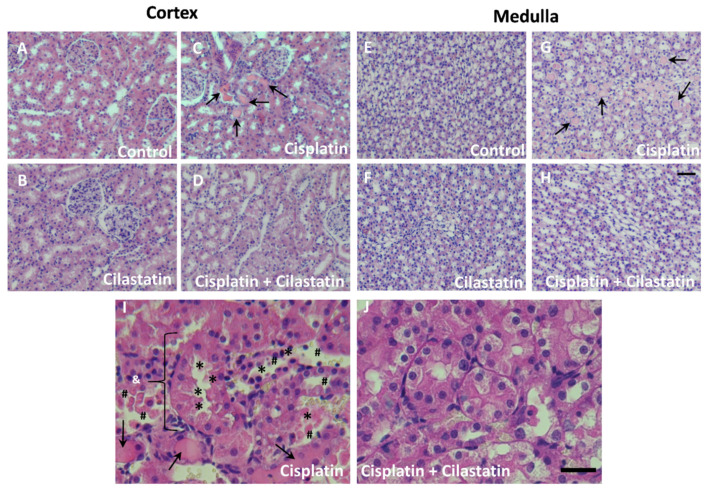
Histopathological analysis of hematoxylin/eosin-stained renal sections shows cilastatin protection against cisplatin-induced damage. Images are shown at 20× magnification for (**A**) control cortex; (**B**) cilastatin cortex; (**C**) cisplatin cortex; (**D**) cisplatin + cilastatin cortex; (**E**) control medulla; (**F**) cilastatin medulla; (**G**) cisplatin medulla; and (**H**) cisplatin + cilastatin medulla. Scale bar from A to H represents 100 µm. Detailed images of renal tubules at 60× magnification are displayed for (**I**) cisplatin and (**J**) cisplatin + cilastatin. Scale bar for I and J represents 25 µm. Renal damage indicators are represented: →: accumulation of protein casts in the renal tubules. & region: tubule swelling. *: loss of brush border membrane. #: cell debris detachment.

Histopathological analysis was also performed in hematoxylin/eosin-stained sections to study morphological alterations related to renal damage. Figure 1 shows cisplatin-induced signs of damage in the proximal renal tubules, including tubule swelling, cell debris detachment, or loss of brush border membrane (Figure 1C,G,I). Protein casts’ accumulation was also observed in cortex and medulla tubules (Figure 1C,G, respectively). This is in contrast to the normal morphology observed in control or cilastatin groups in the cortex (Figure 1A,B, respectively) and medulla (Figure 1E,F, respectively). Cilastatin co-treatment with cisplatin resulted in the clear reduction of all these cisplatin-induced alterations in the cortex and medulla (Figure 1D,H,J).

### 2.2. Cisplatin Alteration of Global Renal Lipids Classes in the Cortex and Medulla. Protective Effect of Cilastatin

**Figure 2:**
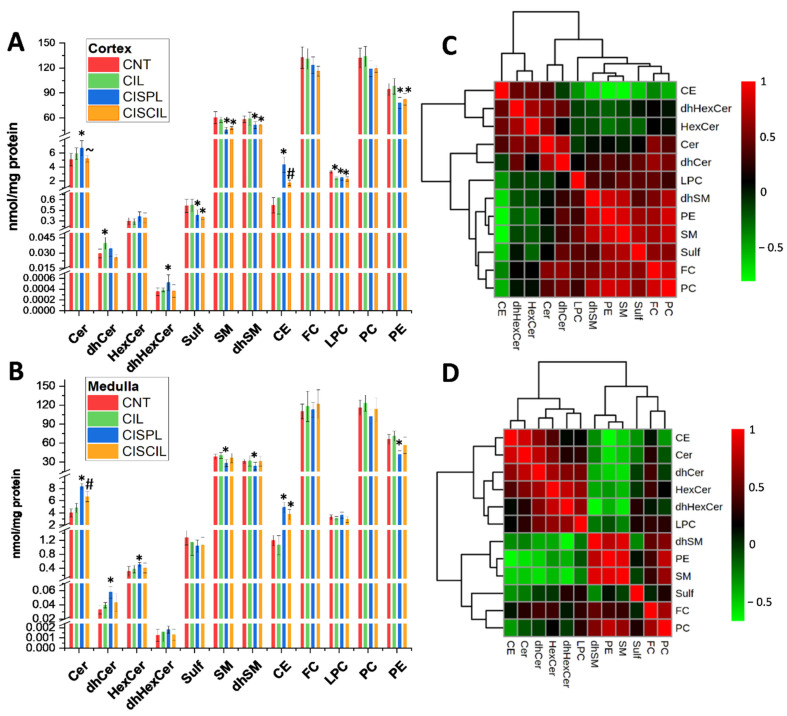
Global effect of cisplatin and cilastatin on renal lipids. Total levels of lipid classes for control (CNT), cilastatin (CIL), cisplatin (CISPL), and cisplatin + cilastatin (CISCIL) groups are presented in the renal (**A**) cortex and (**B**) medulla. Correlation heatmaps are displayed for lipid classes in the (**C**) cortex and (**D**) medulla. * *p* < 0.05 vs. CNT. ~ *p* < 0.05 vs. CISPL # *p* < 0.05 vs. CNT and CISPL. False discovery rate (FDR) < 0.1 was used. Data are presented as the mean ± sd; *n* = 6 animals per group.

Total levels of different lipid classes, including sterols (CE, FC), phospholipids (LPC, PC, PE), and sphingolipids (Cer, dhCer, HexCer, dhHexCer, Sulf, SM, dhSM) were determined in the renal cortex and medulla, in order to evaluate the global effect of cisplatin on lipids during AKI. Complete results on lipid analysis are found in Table S1. As can be seen in Figure 2A,B, quite similar trends were observed in the cortex and medulla for all of the groups. CE and Cer were significantly increased, while SM, dhSM, and PE were decreased, both in cortex and medulla during cisplatin treatment, with respect to control groups. Among these, Cer and CE suffer the most prominent cisplatin-induced changes observed. Moreover, dhCer and HexCer were significantly increased in the medulla, while in the cortex, dhHexCer was increased and LPC and Sulf were decreased during cisplatin treatment.

Co-administration of cilastatin and cisplatin tended to attenuate some of these cisplatin-induced lipid changes, resulting in no difference with respect to control groups in the case of Cer and dhHexCer levels in the cortex, or dhCer, HexCer, SM, dhSM, and PE levels in the medulla. Furthermore, in the case of cisplatin-induced CE increase in the cortex, cilastatin co-administered with cisplatin led to significantly decreased levels compared with the cisplatin group, although the recovery was partial with respect to control groups. The rest of the lipid classes altered by cisplatin remained unrecovered during cilastatin co-treatment. On the other hand, cilastatin itself had no effect on cortex and medulla lipids, except for LPC and dhCer in the cortex, which seemed to be decreased and increased, respectively, compared with the control group. Total levels of FC, PC, and dhHexCer showed no significant change in the cortex and medulla after cisplatin treatment.

Figure 2C,D displays the correlation heatmaps for lipid classes in the cortex and medulla, respectively, offering similar trends.

### 2.3. Individual Renal Lipid Species Alteration by Cisplatin Treatment. Cilastatin Attenuating Effect

#### 2.3.1. Cholesterol Esters

**Figure 3:**
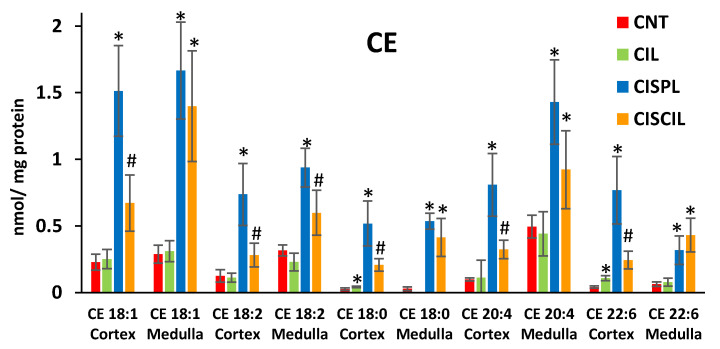
Cholesterol ester (CE) species found in the renal cortex and medulla in CNT, CIL, CISPL, and CISCIL groups. * *p* < 0.05 vs. CNT. # *p* < 0.05 vs. CNT and CISPL. FDR < 0.1 was considered. Data are presented as the mean ± sd; *n* = 6 animals per group.

Analysis of five individual CE species (CE 18:1, CE 18:2, CE 18:0, CE 20:4, and CE 22:6) carried out in the renal cortex and medulla revealed that cisplatin significantly increased each of them compared with control levels (Figure 3). On the other hand, co-administration of cilastatin and cisplatin led to a general decrease in individual CE species levels with respect to cisplatin treatment, which was statistically significant for all the CE species in cortex and CE 18:2 in medulla, as can be seen in Figure 3. This recovery in cortex CE species due to cilastatin co-treatment was partial compared with control group levels, while no statistically significant recovery in most medulla CE species (except for CE 18:2) was observed for cilastatin co-administration compared with cisplatin. Complete statistical analysis results on cortex and medulla lipid species are shown in Tables S2 and S3, respectively.

#### 2.3.2. Sphingolipids: Cer, dhCer, HexCer, dhHexCer, SM, dhSM, and Sulf

**Figure 4:**
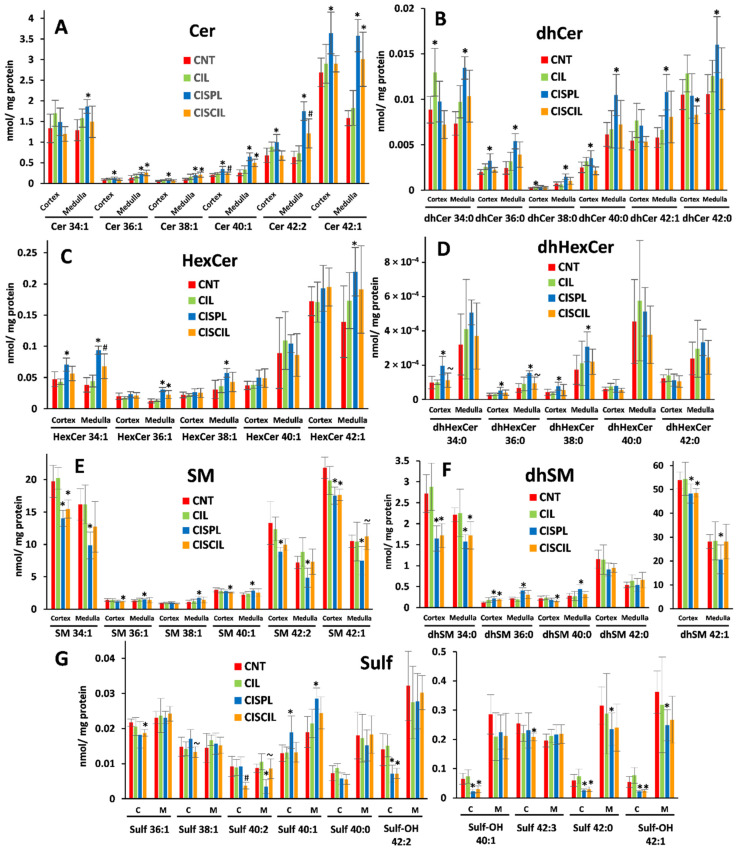
Sphingolipid species found in the renal cortex and medulla in CNT, CIL, CISPL, and CISCIL groups. (**A**) Ceramides (Cer). (**B**) Dihydroceramides (dhCer). (**C**) Hexosylceramides (HexCer). (**D**) Dihydrohexosylceramides (dhHexCer). (**E**) Sphingomyelins (SM). (**F**) Dihydrosphingomyelins (dhSM). (**G**) Sulfatides (Sulf). * *p* < 0.05 vs. CNT. ~ *p* < 0.05 vs. CISPL. # *p* < 0.05 vs. CNT and CISPL. FDR < 0.1 was considered. Data are presented as the mean ± sd; *n* = 6 animals per group.

A total of 43 sphingolipid species were quantified in the renal cortex and medulla to evaluate the impact of cisplatin and cilastatin treatment on their levels, as displayed in Figure 4. Regarding Cer (Figure 4A), dhCer (Figure 4B), HexCer (Figure 4C) and, dhHexCer (Figure 4D) species, as can be seen, cisplatin treatment resulted in a significant increase in 19 species from these four classes in the renal cortex and medulla. Again, co-treatment with cisplatin and cilastatin shows a tendency to attenuate the effect of cisplatin, with a general decrease in the cisplatin-altered lipid levels in the cortex and medulla. In fact, for dhCer (Figure 4B) and dhHexCer (Figure 4D) species, no difference was observed between cisplatin + cilastatin treated groups and control groups for those cisplatin-altered species, indicating a full recovery. In the case of Cer (Figure 4A) and HexCer (Figure 4C), this recovery was also the case for some altered species, such as HexCer 34:1 (cortex and medulla) and HexCer 38:1 and HexCer 42:1 (medulla); Cer 36:1, Cer 38:1, Cer 40:1, Cer 42:1, and Cer 42:2 in the cortex; and Cer 34:1 and Cer 42:2 in the medulla, being either totally or partially recovered. Conversely, cisplatin + cilastatin treatment still showed significant differences with respect to control groups for Cer 36:1, Cer 38:1, Cer 40:1, Cer 42:1, and HexCer 36:1 in the medulla, with no recovery, pointing to a much better recovery effect exerted by cilastatin in the cortex than in the medulla for Cer and HexCer.

In the case of SM (Figure 4E) and dhSM (Figure 4F) species, cisplatin led to a decrease in the cortex and medulla for the shortest and longest chain species, which was statistically significant for SM 34:1, dhSM 34:0, SM 42:2, SM 42:1, and dhSM 42:1. In contrast, the effect observed for cisplatin treatment on intermediate chain SM and dhSM species in the cortex and medulla was an increase, which was statistically significant for dhSM 36:0 (both in cortex and medulla) and dhSM 40:0, SM 36:1, SM 38:1, and SM 40:1 in the medulla. Co-administration of cisplatin and cilastatin tended to normalize SM and dhSM species levels in many cases, with SM 34:1, SM 36:1, SM 38:1, SM 40:1, SM 42:2, SM 42:1, dhSM 36:0, dhSM 40:0, and dhSM 42:1 showing no difference with respect to control groups in the medulla. Finally, some Sulf species were also altered by cisplatin treatment in the renal cortex and medulla, as shown in Figure 4G. While Sulf 40:1 was found to be significantly increased in both the cortex and medulla after cisplatin treatment, other species such as Sulf-OH 40:1 or long-chain Sulf-OH 42:2, Sulf-OH 42:2 were decreased in the cortex, with Sulf 42:0 and Sulf-OH 42:1 also being decreased in both the cortex and medulla. Cilastatin co-administered with cisplatin led again to a mild recovery effect, especially for Sulf 40:1 (cortex and medulla) and Sulf 40:2 and Sulf-OH 42:1 (medulla), showing no difference compared with control groups.

#### 2.3.3. Phospholipids: PC, PE, and LPC

**Figure 5:**
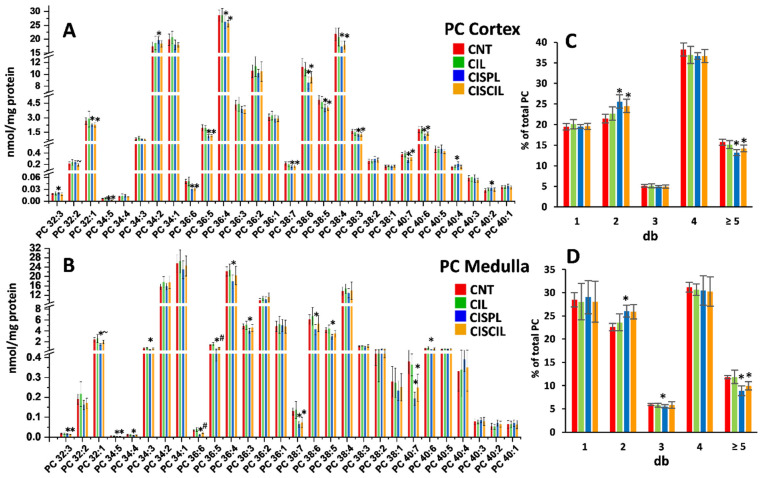
Phosphatidylcholine (PC) species found in the (**A**) cortex and (**B**) medulla in CNT, CIL, CISPL, and CISCIL groups. Percentage of PC species containing different double bonds are presented in the (**C**) cortex and (**D**) medulla. * *p* < 0.05 vs. CNT. ~ *p* < 0.05 vs. CISPL. # *p* < 0.05 vs. CNT and CISPL. FDR < 0.1 was considered. Data are presented as the mean ± sd; *n* = 6 animals per group.

Quantification of 60 phospholipid species (28 PC, 20 PE, and 12 LPC) was carried out in the renal cortex and medulla for the assessment of potential cisplatin-induced alterations and cilastatin renoprotective effect. The results for PC species are shown in Figure 5A,B, respectively. As can be seen, cisplatin significantly altered up to 19 PC species in either the cortex or medulla. Most of these PC species were decreased by cisplatin, with the exception of PC 40:2, PC 40:4, and PC 34:2, which were increased in the cortex. The fraction of highly unsaturated PC species was significantly decreased in both the medulla and cortex, while the fraction of species containing two double bonds was significantly increased, as can be seen in Figure 5C,D. Treatment with both cisplatin and cilastatin showed similar effects to those previously observed, with some attenuating effect observed, in some cases leading to a full recovery of PC levels, with no significant differences with the control group, or to a partial recovery. This recovery was mainly observed in the renal medulla.

**Figure 6:**
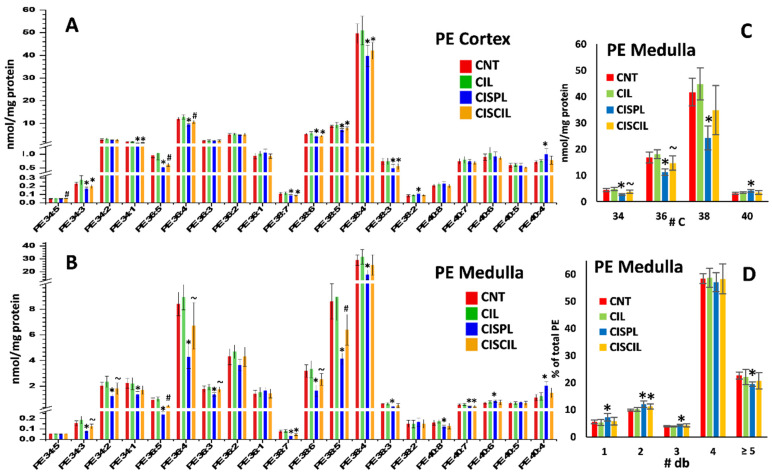
Phosphatidylethanolamine (PE) species found in the renal (**A**) cortex and (**B**) medulla in CNT, CIL, CISPL, and CISCIL groups. The amount of PE species containing different total length of fatty acid chains (**C**) and percentage of PE species containing different double bonds (**D**) are presented in medulla. * *p* < 0.05 vs. CNT. ~ *p* < 0.05 vs. CISPL. # *p* < 0.05 vs. CNT and CISPL. FDR < 0.1 was considered. Data are presented as the mean ± sd; *n* = 6 animals per group.

The results for PE species determination in the cortex and medulla are shown in Figure 6A,B, respectively. A total of 16 PE species were significantly altered by cisplatin in either the cortex or medulla, with the highest number of changed species found in the medulla. In most of the cases, PE species were decreased by cisplatin treatment, except for PE 38:2, PE 40:6, and PE 40:4, which were increased either in the cortex, the medulla, or both the cortex and medulla, respectively. Accordingly, considering the total length of fatty acid chains in the PE species in the medulla, the total amount of PE with 34, 36, and 38 C was significantly decreased with cisplatin, while species of 40 C were increased, as depicted in Figure 6C. Cilastatin co-treatment with cisplatin attenuated most of the cisplatin-induced changes in individual PE species found in the medulla, offering levels with no significant differences compared with control groups in 11 out of 15 cisplatin-altered species in medulla, with 2 more PE species partially recovered. However, in the cortex, only 2 out of 11 cisplatin-altered species were recovered with cilastatin to control levels, with 2 additional PE species being partially recovered. Similar to the observations for PC species, the fraction of highly unsaturated PE species was significantly decreased in the medulla, while the fraction of species containing 1–3 double bonds was significantly increased, as displayed in Figure 6D.

**Figure 7:**
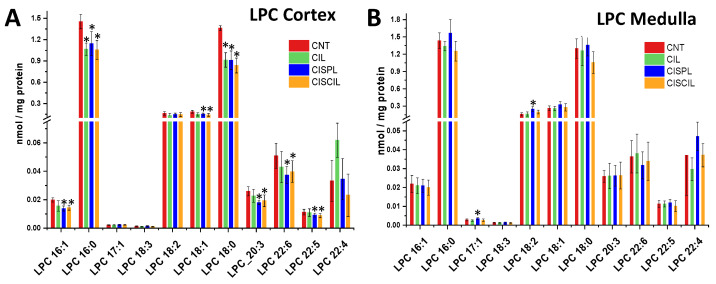
Lysophosphatidylcholine (LPC) species found in the renal (**A**) cortex and (**B**) medulla in CNT, CIL, CISPL, and CISCIL groups. * *p* < 0.05 vs. CNT. Data are presented as the mean ± sd; *n* = 6 animals per group.

Regarding LPC species analysis, most of the cisplatin-induced changes observed were found in the renal cortex, where LPC 16:1, LPC 16:0, LPC 18:1, LPC 18:0, LPC 20:3, LPC 22:6, and LPC 22:5 were significantly decreased with respect to control samples, as can be seen in Figure 7A. On the other hand, LPC 17:1 and LPC 18:2 were found to be increased in the medulla during cisplatin treatment (Figure 7B). Co-treatment with cisplatin and cilastatin had no recovery effect on cortex LPC species. Conversely LPC 17:1 and LPC 18:2 were recovered in the medulla, with no significant differences compared with control samples.

### 2.4. Renal Lipids as Classification Variables for Kidney Damage and Protection

**Figure 8:**
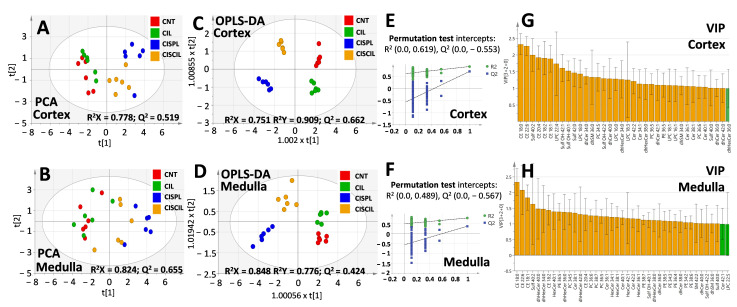
Multivariate analysis of cortex and medulla lipids for simultaneous comparison of CNT, CIL, CISPL, and CISCIL groups. Principal component analysis (PCA) scores plots for (**A**) cortex lipids and (**B**) medulla lipids. Orthogonal partial least squares discriminant analysis (OPLS-DA) score plots for (**C**) cortex and (**D**) medulla lipid variables. Here, 100 cycle permutation tests were done for OPLS-DA models validation using (**E**) cortex and (**F**) medulla lipids. Variable importance in projection (VIP) scores, for the OPLS-DA models, higher than 1 are presented in orange for most relevant (**G**) cortex and (**H**) medulla lipids influencing groups separation.

Multivariate analysis was performed using all of the lipid variables measured in the cortex and medulla to assess whether these species could serve for group classification and discerning cisplatin-induced renal damage and nephroprotection by cilastatin. Principal component analysis (PCA) and orthogonal projections to latent structures discrimination analysis (OPLS-DA) were carried out using separately either cortex or medulla lipids, as displayed in Figure 8. PCA analysis of cortex (Figure 8A) and medulla (Figure 8B) lipids offered models the ability to show a clear separation of the cisplatin group with respect to control and cilastatin groups, the latter two clustering closely. On the other hand, the cisplatin + cilastatin treated group tended to separate from the cisplatin group and located in between the control and cisplatin groups, suggesting differences between the groups. All the PCA models presented R^2^ higher than 0.7 and Q^2^ values higher than 0.5.

Multi-group OPLS-DA analysis also showed clearly separated clustering of the different groups both using cortex (Figure 8C) or medulla (Figure 8D) lipid variables. OPLS-DA models presented acceptable Q^2^ values and R^2^ values higher than 0.7 and proved to be valid during permutation testing for 100 iterations, as shown in Figure 8E,F. Features with variable importance in the projection (VIP) scores higher than 1 can be found in Figure 8G,H, indicating cortex or medulla lipids, respectively, which have a higher impact on the groups’ separation. These comprised diverse lipid species from the CE, sphingolipid, and phospholipid classes.

**Figure 9:**
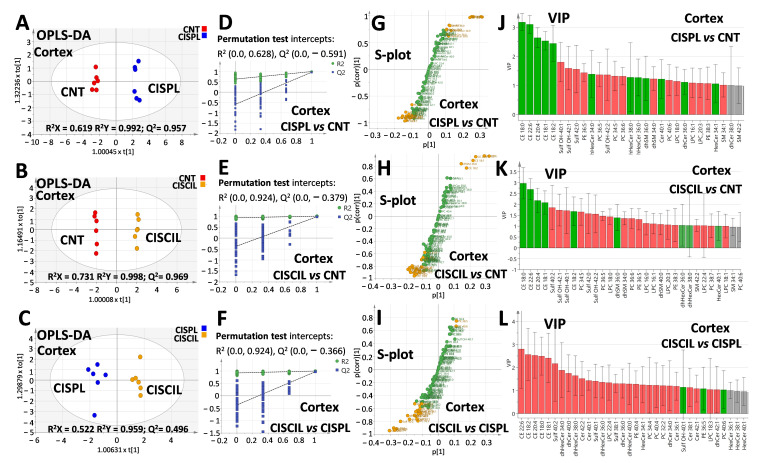
OPLS-DA analysis for renal cortex lipids. Score plots for (**A**) CISPL vs. CNT groups, (**B**) CISCIL vs. CNT groups, and (**C**) CISCIL vs. CISPL groups. Permutation tests based on 100 iterations for the models for (**D**) CISPL vs. CNT, (**E**) CISCIL vs. CNT, and (**F)** CISCIL vs. CISPL. S-plots are presented with those features with both |p(corr)| > 0.5 and VIP > 1 marked in orange for (**G**) CISPL vs. CNT, (**H**) CISCIL vs. CNT, and (**I**) CISCIL vs. CISPL. VIP plots are presented in green and red for features with a value >1, with an increasing and decreasing trend, respectively; for (**J**) CISPL vs. CNT, (**K**) CISCIL vs. CNT, and (**L**) CISCIL vs. CISPL.

**Figure 10:**
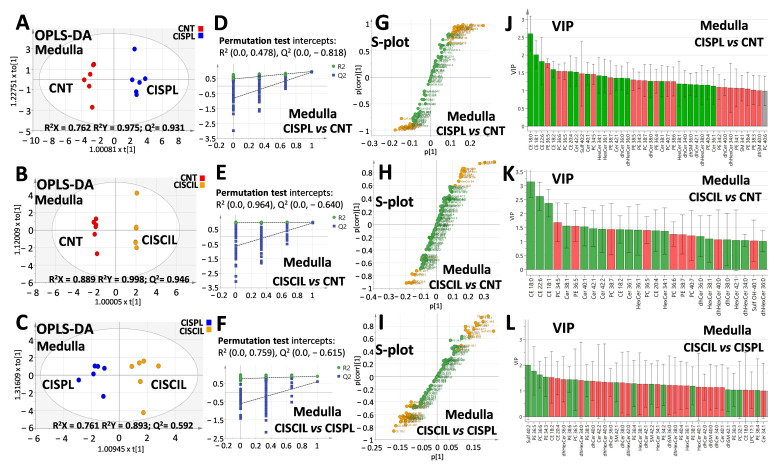
OPLS-DA analysis for renal medulla lipids. Score plots for (**A**) CISPL vs. CNT, (**B**) CISCIL vs. CNT, and (**C**) CISCIL vs. CISPL. Permutation tests based on 100 iterations for the models for (**D**) CISPL vs. CNT, (**E**) CISCIL vs. CNT, and (**F**) CISCIL vs. CISPL. S-plots are presented with those features with both |p(corr)| > 0.5 and VIP > 1 marked in orange for (**G**) CISPL vs. CNT, (**H**) CISCIL vs. CNT, and (**I**) CISCIL vs. CISPL. VIP plots are presented in green and red for features with a value >1, with an increasing and decreasing trend, respectively; for (**J**) CISPL vs. CNT, (**K**) CISCIL vs. CNT, and (**L**) CISCIL vs. CISPL.

To determine specific lipid differences between individual groups related to cisplatin treatment and nephroprotection, further OPLS-DA analysis was performed separately with cortex and medulla lipids, as can be seen in Figure 9 and Figure 10, respectively. OPLS-DA models were built for cisplatin compared with the control group, cisplatin + cilastatin compared with cisplatin, as well as cisplatin + cilastatin compared with the control group. The most discriminant OPLS-DA models were the ones between control and either cisplatin- or cisplatin + cilastatin-treated groups, considering their highest R^2^ and Q^2^ values, with proper validation based on permutation test results with 100 cycles (Figure 9A,B,D,E and Figure 10A,B,D,E).

On the other hand, OPLS-DA models for cisplatin + cilastatin and cisplatin groups, both using cortex (Figure 9C) or medulla (Figure 10C) lipids, also allowed group discrimination, with reasonable R^2^ and Q^2^ values and appropriate validation results from 100-cycle permutation tests (Figure 9F and Figure 10F), with medulla lipids being those presenting better predictability. S-plots for the different OPLS-DA models are included in Figure 9G,H,I and Figure 10G,H,I, for either cortex or medulla lipid features. Species with a |p(corr)| higher than 0.5 and VIP scores higher than 1 have been marked in the S-plots as the most relevant features for groups’ discrimination.

Complete data including p(corr) and VIP values from the OPLS-DA models, along with the univariate statistics for each lipid species, can be found in Supplementary Tables S2 (cortex lipids) and S3 (medulla lipids). It seems clear that renal cortex and medulla lipids can serve as discriminating criteria for cisplatin-induced renal damage and for cilastatin nephroprotection during cisplatin treatment.

### 2.5. Renal Lipids as Potential Biomarkers of Cisplatin-Induced Renal Damage

In order to select potential lipid biomarkers of renal damage caused by cisplatin, a multiple criteria combination of statistically significant *p*-values from univariate analysis of cisplatin versus control groups, as well as p(corr) and VIP values found in the corresponding OPLS-DA model, was employed. Selected lipids with *p*-values < 0.05; FDR < 0.1; |p(corr)| > 0.5 and VIP > 1 can be found in Table 2 (cortex lipids) and Table 3 (medulla lipids), where fold change (FC) and areas under the curve (AUC) for receiver operating characteristic (ROC) curves are also included.

As can be seen, up to 40 lipids from the medulla and 27 lipids from the cortex, comprising CE, sphingolipids, and phospholipid species, are selected in Table 2 and Table 3, respectively. AUC values for ROC curves obtained for each lipid were higher than 0.85 and, in most of cases, close to 1, indicating good discrimination capabilities of the species between cisplatin and control groups. Remarkably, the cisplatin-increased CE species (CE 18:1, CE 18:2, CE 18:0, CE 20:4, and CE 22:6) seem to be the most discriminating features both in the medulla and cortex, showing the highest FC, VIP, p(corr), and AUC values. dhHexCer 34:0 is another increased lipid in the cortex with a high FC and relevant scores. On the other hand, sulfatides such as Sulf 42:0, Sulf-OH 40:1, or Sulf-OH 42:1 are also quite relevant in the cortex, while PE 36:5, PE 38:6, PE 38:5 PC 36:5, or PC 36:6 are also very important in the medulla, all of them being decreased during cisplatin treatment.

### 2.6. Renal Lipids as Potential Biomarkers of Cilastatin Nephroprotection

To select potential lipid biomarkers of cilastatin protection against cisplatin nephrotoxicity, the same multiple criteria approach as in Section 2.5, was applied using univariate analysis of cisplatin + cilastatin versus cisplatin groups, and p(corr) and VIP values found in the corresponding OPLS-DA model. Selected lipids with *p*-values < 0.05; FDR < 0.1; |p(corr)| > 0.5, and VIP > 1 can be found in Table 4 (cortex lipids) and Table 5 (medulla lipids), where FC and AUC for ROC curves were also calculated.

As can be seen in Table 4, all the CE species (CE 18:1, CE 18:2, CE 18:0, CE 20:4, and CE 22:6) in the cortex could be regarded as the most powerful indicators of the protection exerted by cilastatin, significantly reducing, at the same time, the alterations induced by cisplatin. PE 36:5 and dhHexCer 34:0 in the cortex are also important potential biomarkers of cilastatin protection, especially the latter, which undergoes a total recovery owing to cilastatin towards control levels. Regarding medulla lipids, as shown in Table 5, a higher amount of potential biomarkers of cilastatin protection was found than in cortex. Notably, Sulf 40:2 seems to be the most discriminating species in the medulla, considering its highest FC and VIP scores. Diverse PC and PE species were also found, including PE 36:5, PE 38:5, PE 38:6, PE 36:4, PE 34:3, and PC 36:6, to name a few, in addition to Cer 42:2, dhHexCer 36:0, and HexCer 34:1, as significant biomarkers of cilastatin protection in the medulla. In all of the cases, these lipids were associated with either a partial or total cilastatin-induced recovery of control levels compared with their altered levels by cisplatin. For all the species, AUC values for ROC curves obtained were higher than 0.85, proving their good discrimination capabilities.

## 3. Discussion

In this work, we have quantified a total of 108 lipid species comprising important structural lipids such as phospholipids, sphingolipids, and cholesterol along with its esterified forms, on the kidney cortex and medulla from rats treated with cisplatin and/or cilastatin. This was performed in order to better understand the nephrotoxic effect of cisplatin and the protective effect of cilastatin. Up to 63 lipid species were found to be significantly altered in the medulla by cisplatin treatment, while 56 lipid species were affected in the cortex, reflecting renal damage in both regions. Although, traditionally, most cisplatin-induced AKI studies focus on proximal tubule cells and renal cortex damage, where drug accumulation is mainly focused, recent studies point to the fact that injury in the medulla may even be as severe as in the cortex, based on TUNEL apoptosis assays [5]. This might be related to the accumulation of cisplatin in segment S3 of proximal tubules [3,5], descending towards the outer medulla, aside from the potential direct and secondary damage of Henle’s loop [4]. In addition, the medulla was pointed out as the most sensitive renal area to observe cisplatin-related global metabolite changes as compared with the cortex [5]. Our previous results also pointed to a direct damage of medulla cells (aside from secondary damage) by cisplatin, reflected in diverse phospholipid and cardiolipin alterations [4]. These new results expand those observations with additional lipid species and classes altered by cisplatin and reinforce the relevance of medulla damage in cisplatin-induced AKI.

Although FC in kidney was not significantly altered by cisplatin treatment, both total CE and all the measured individual CE species were remarkably increased in both the cortex (eightfold increase for total CE) and medulla (fourfold increase for total CE) in association with cisplatin-induced renal damage. Cholesterol is a key component of plasma membranes, interlocating among phospholipids and influencing fluidity, surface charge, and interactions of polar head groups [35,36]. Its presence is also fundamental in lipid rafts, establishing hydrophobic interactions with SM, and being essential for segregation of molecules and to control their interaction with other components in the membrane [37]. Cells maintain a cholesterol balance between de novo synthesis in the endoplasmic reticulum (ER) and LDL-mediated uptake. Most cholesterol is located in the plasma membrane, but can also cycle back to the ER where FC can be converted (through ACAT-mediated esterification) into CE, which is considered as a cytosolic storage form of cholesterol [36]. CE can also be hydrolyzed back to FC, constituting the cholesterol ester cycle [36]. The fact that FC was unaltered during cisplatin treatment, but CE was increased in both the cortex and medulla, suggests an altered renal cholesterol metabolism associated with cisplatin-induced AKI. Previous studies showed quite similar observations in both HK-2 human proximal tubule cells and in the renal cortex from mice, during acute ischemic kidney injury, with unaltered FC and increased CE levels [36]. In that case, plasma membrane injury, triggering increased FC trafficking from the membrane to the ER, seemed to be the most plausible explanation, resulting in an escalating CE generation. In this regard, damage to cholesterol-rich lipid rafts rather than plasma membrane appeared to be more effective in the increased cholesterol trafficking to ER [36]. A cytoprotective role of CE has also been implied in such previous studies. An increase in CE levels was also reported in the rat renal cortex during early stage diabetic nephropathy [29]. The results are also in agreement with a global increase in neutral lipids in renal proximal tubules reported during cisplatin treatment [32]. Our results point for the first time to a global increase in total CE in addition to individual CE species, both in the renal cortex and medulla, during cisplatin-induced AKI. Cilastatin itself, on the other hand, seemed to slightly increase CE 18:0 and CE 22:6 only in the cortex, although total levels of FC or CE were unaltered. This slight effect could be due to the fact that its target, DHP-I, is located in the lipid rafts in the proximal tubule (mainly in the cortex), and some small disturbance of the membrane might be implied during its interaction. Co-administration of cisplatin and cilastatin, on the other hand, led to a reduction in all the cisplatin-increased CE species in the cortex, while in the medulla, almost no recovery effect was observed, with only a partial recovery of CE 18:2. This points to the specific action of cilastatin in the proximal tubule cells, exerting a significant protection of cell death expansion related to direct damage of cisplatin on plasma membrane and lipid rafts in the cortex, while cell damage related to cholesterol imbalance in the whole medulla seems to remain largely unprotected.

Sphingolipids are active lipids mainly found in the cell membranes and are quite rich in lipid rafts [37]. Ceramides are the central metabolites of sphingolipid metabolism, involving diverse molecules and enzymes with important roles in cellular processes, including cell growth, apoptosis, differentiation, drug resistance, and senescence [38,39]. Additionally, sphingolipids participate in the pathways related to cisplatin cancer cell death and AKI [33]. Ceramides can be generated by several pathways, including de novo synthesis, hydrolysis of sphingomyelin, or recycling of complex sphingolipids [38]. De novo synthesis is the main route, and takes place in the ER, from palmitoyl-CoA and serine, through a series of enzymatic pathways to produce sphinganine, which leads to dihydroceramides through dihydroceramide synthase (CerS). Finally, desaturation of dhCer generates Cer [40]. SM can also be hydrolyzed by sphingomyelinases, producing Cer. Cer can be transformed into more complex sphingolipids in the Golgi apparatus: phosphorylated to generate Cer-1-P, a signaling molecule; converted to SM by the action of SM synthase; or glycosylated to produce hesoxylceramides (glucosyl- or galactosyl- ceramides), precursors of sulfatides [38]. Cer can also be degraded to sphingosine, a precursor of sphingosine-1-phosphate (S1P), with important roles in cell survival and proliferation, inflammation, and drug resistance [39]. Finally, degradation of S1P to ethanolamine and hexadecenal is the exit pathway of this route [38].

During quantification of sphingolipid species, a global increase in the total levels of Cer, dhCer, HexCer, and dhHexCer was observed during cisplatin treatment, which was especially relevant for Cer. Differences with respect to control kidney were more obvious when individual subspecies were quantified, with 19 out of 22 species being increased either in the cortex (11 species) and/or medulla (18 species) with cisplatin treatment. This is in agreement with previous observations pointing to an increase in Cer and HexCer in the mouse renal cortex associated with cisplatin treatment [33], but herein, we have found that the medulla is also affected by this effect.

These observations can reflect an increased metabolic production of Cer/dhCer and HexCer/dhHexCer. On the one hand, this could take place through increased CerS activity, in light of previous observations in the renal cortex, where long-chain CerS activity was measured during cisplatin treatment and inhibition of CerS led to a reduction in the observed Cer/HexCer accumulation [33]. Moreover, a cisplatin-induced reduction in SM/dhSM levels found in the cortex and medulla suggests an additional increased production of Cer/dhCer by hydrolysis of SM/dhSM through the sphingomyelinase (SMase) pathway [38], with an special impact on long-chain Cer/dhCer, all being detrimental in models of AKI [40]. This would also be in agreement with the fact that long-chain Cer species seem to be relevant during AKI [40] and with previous observations of an increased SMase activity in the renal cortex during cisplatin treatment [33]. Cer is involved in cisplatin-induced apoptosis via signaling in both intrinsic and extrinsic pathways [39]. In the intrinsic mitochondrial pathway, cisplatin causes mitochondrial outer membrane permeabilization. The increase in Cer, along with its downstream metabolites, can contribute to this permeabilization, and the channels’ formation by Cer facilitates apoptosis in the mitochondrial membrane, allowing the entry of Bax into the mitochondrial outer membrane, as well as the outflow of cytochrome C [39]. Furthermore, Fas-mediated extrinsic apoptosis employs Fas receptor located in the lipid rafts, with its clustering being mediated by cisplatin-caused SMase activation and Cer elevation [39]. Therefore, Cer determines apoptosis in cisplatin-induced AKI, while their leveling via their transformation to HexCer seems to be protective [33] and is associated with resistance to cisplatin [39]. Cer generated in great amounts can also affect the physical properties of membranes and can displace cholesterol and drive its esterification [35]. On the other hand, SM is mainly present in plasma membranes and has a direct association with cholesterol molecules, especially important in lipid rafts [41]. A lack of SM regeneration could be negative for proper membrane function [41].

Sulf are abundant in the kidney, especially in the apical membrane of distal tubules [42]. Although they are not essential for normal kidney function, a deficiency of Sulf has been found to be compensated by increased generation of sulfated glycolipids and cholesterol [42]. Sulfatide is a key L-selectin ligand in the kidney, with a vital role in monocyte infiltration into the kidney interstitium [42]. On the other hand, lipid-raft associated myelin and lymphocyte protein (MAL) forms complexes with sulfatides and glycosphingolipids in renal membranes, contributing to stabilization and sorting of glycosphingolipid-enriched microdomains [42]. Our results on Sulf alterations in the cortex and medulla confirm previous studies where diverse sulfatide species were also thought to be altered by cisplatin in the kidney [34].

All the Cer, dhCer, HexCer, and dhHexCer species altered in the cortex by cisplatin were partially or totally recovered by cilastatin co-administration, while in the medulla, a large amount of Cer species were not recovered, with an almost complete recovery of dhCer, HexCer, and dhHexCer. Considering the involvement of Cer species in Fas-mediated extrinsic apoptosis and its blockage by cilastatin binding to DHP-I in lipid rafts at the proximal tubule, this could explain the protective effect and Cer recovery taking place mainly in cortex cells. As for SM, dhSM, and Sulf, cilastatin recovery seemed to be more relevant in the medulla than in the cortex, suggesting that secondary damage is impaired by cilastatin action in proximal tubules reaching the outer medulla, where most renal damage is concentrated [4].

Phospholipids are the most abundant species in cell membranes, where PC and, to a lesser extent, PE predominate, with a higher presence of PC in the luminal side and PE being more abundant in the cytosolic leaflet [35,43]. Lysophospholipids are, on the other hand, signaling species that can be generated from hydrolysis of glycerophospholipids including LPC [35]. Most of the 60 PC, PE, and LPC species quantified were found to be altered by cisplatin treatment, either in the cortex or medulla, with PC and LPC presenting a higher amount of changes in the cortex, while PE-associated changes were more numerous and prominent in the medulla. Alterations of renal PC, PE, or LPC species as a result of cisplatin treatment are in accordance with previous reported observations in kidney during AKI [4,5,26,34,44]. Our results indicate that most altered PE, PC, and LPC species were decreased by cisplatin in both the cortex and medulla. Regarding the cilastatin nephroprotective effect, strikingly, a higher degree of partial or total recovery effect was observed in the medulla for cisplatin-altered lipids than in the cortex. Another interesting fact is that all those cisplatin-increased PC, PE, and LPC species in the cortex and medulla were recovered by cilastatin, while most of cisplatin-decreased species in the cortex were still altered during co-treatment with cilastatin. This might indicate a higher degree of direct damage caused by cisplatin accumulation in the cortex, remaining unprotected by cilastatin, and leading to cell death and associated PC, PE (the main components of the cell membrane), and LPC release in apoptotic bodies and, therefore, the reduction in their levels. In contrast, secondary damage associated with extrinsic apoptosis and consequent protein cast accumulation in proximal tubules can be protected by cilastatin. This would imply that PC, PE, and LPC species decreased in the medulla and are more related to cisplatin-driven secondary cell damage. On the other hand, increased PC, PE and LPC species might be associated with either cell membrane regeneration or signaling processes, which, according to these results, might be more related to secondary extrinsic apoptosis cisplatin-caused damage protected by cilastatin.

In addition, a cisplatin-induced decrease in the percentage of PC and PE species with highly unsaturated fatty chains was also observed, which can be related to lipid peroxidation processes, and would be reduced by cilastatin [4,10,11].

Globally, within the lipid species herein quantified, the recovery effect of cilastatin was observed in 26 out of 56 cisplatin-altered lipids in the cortex, and in 50 out of 63 cisplatin-altered lipids in the medulla. Perhaps, this reflects a high degree of lipid changes associated with unprotected direct mitochondrial damage exerted by cisplatin mainly in the cortex compared with the medulla, with more lipid changes associated with secondary damage protection detected in the whole medulla.

Moreover, multivariate analysis allowed the identification of potential new lipid biomarkers for both cisplatin-induced AKI and cilastatin nephroprotection, including CE, Sulf, PE, PC, Cer, or HexCer species, as described in Section 2.5 and Section 2.6. From our findings, CE 18:2 and PE 36:5 are possible candidate biomarkers able to discern at the same time cisplatin damage and cilastatin nephroprotection in both the cortex and medulla. However, as the recovery of these two species with cilastatin is partial, more appropriate species as biomarkers would be dhHexCer 34:0 in the cortex and Sulf 42:0 in the medulla, being able to distinguish both renal damage and protection, with cilastatin fully recovering their cisplatin-altered levels to those of the control group. This could be extrapolated to any Fas/Fas ligand-mediated renal injury, but should be confirmed in future studies. Several new biomarkers of AKI have been proposed in recent years, mainly proteins (e.g., KIM-1 [45] or NGAL [46]). The fact that we have found potential new biomarkers of cisplatin-induced AKI and associated cilastatin protection, of lipid nature in kidney tissue, expands on the existing knowledge and provides complementary options for a better diagnosis, prognosis, and follow-up. In fact, a combination of biomarkers’ detection might even potentially be used as an indication of a specific AKI caused either by a particular nephrotoxicant (such as cisplatin) or another cause, and even distinguish disease state/progression. Another step further would be determining if our results on altered renal lipid patterns are also reflected in circulating lipids in blood or in urine. This would make biomarker detection for cisplatin-induced AKI and cilastatin nephroprotection more feasible in patients.

Cisplatin-induced AKI-related changes in lipid levels are numerous and diverse, comprising important structural lipids at the complete renal structure: the cortex and medulla. Attenuation of many of these changes by cilastatin shows its great potential for improving renal function and reducing lipid-related structural changes, in correlation with a normalized renal morphology. Again, cilastatin has proved useful for discerning unprotected direct cell damage caused by cisplatin and secondary changes related to primary damage, which can be impaired in the proximal tubules by cilastatin blockage of the Fas-mediated apoptotic pathway.

## 4. Materials and Methods

### 4.1. Reagents

Cilastatin (kindly provided by Merck Sharp and Dohme S.A., Madrid, Spain) and cisplatin (Pharmacia Nostrum (Madrid, Spain) were employed. A 0.9% NaCl solution (Braun Medical S.A, Barcelona, Spain) was used for preparation of drug solutions for administration.

N-dodecanoyl-D-erythro-sphingosylphosphorylcholine [SM 30:1 (d18:1/12:0)], D- glucosyl-ß-1,1′-N-dodecanoyl-D-erythro-sphingosine [HexCer 30:1 (d18:1/12:0)], N-dodecanoyl-D-erythro-sphinganylphosphorylcholine [dhSM 30:0 (d18:0/12:0)], 1,2-dimyristoleoyl-sn-glycero-3- phosphocholine [PC 28:2 (14:1/14:1)], 1-heptadecanoyl-2-hydroxy-sn-glycero-3-phosphocholine [LPC (17:0)], and 1,2- dipalmitoleoil-sn-glycero-3-phosphoethanolamine [PE 32:2 (16:1/16:1)] were purchased from Avanti Polar Lipids (Alabaster, Alabama, USA). Deuterated N-tetracosanoyl-D-erythro-sphingosine [Cer 42:1-d7 (d18:1/24:0)], N-stearoyl-D-erythro-dihydrosphingosine [dhCer (36:0-d3)], and C16-3′-sulfogalactosylceramide [Sulf 34:1(d18:1/16:0)] were acquired from Matreya LLC (State College, PA, USA). Cholesterol-d7 (FC-d7) and cholesteryl-d7 palmitate [CE (16:0-d7)] were from Sigma-Aldrich.

HPLC or LC-MS grade solvents and reagents were exclusively employed, including methanol (MeOH), acetonitrile (ACN), and isopropanol (iPrOH) (VWR International Eurolab, Barcelona, Spain), as well as chloroform, dichloromethane, and ammonium formate (NH_4_COOH) (Sigma-Aldrich, Merck Life Science S.L., Madrid, Spain). Ultrapure water was obtained from a Milli-Q purification system (Millipore, Merck Life Science S.L., Madrid, Spain).

### 4.2. Animal Model

Adult male wistar rats (WKY, Criffa, Barcelona, Spain) were bred at the Instituto de Investigación Sanitaria Gregorio Marañón (IiSGM, Madrid, Spain). These were kept under controlled temperature, light, and humidity conditions with free access to food and water. Treatments were administered intraperitoneally as previously described [4,11] to four groups of rats (*n* = 6 animals per group): Group 1: Control (CNT)—0.9% NaCl was injected to the rats in the same fashion as treatments for groups 3 and 4; Group 2: Cilastatin (CIL)-injected (150 mg kg^−1^ of body weight (bw) per day); Group 3: Cisplatin (CISPL)-injected (a unique dose of 5 mg kg^−1^ bw at day 0); and Group 4: Cisplatin + cilastatin (CISCIL)-injected 5 mg kg^−1^ bw at day 0 and cilastatin-injected (150 mg kg^−1^ bw per day). Animals were sacrificed five days after the treatment began. Prior to this, 24 h urine was collected in metabolic cages from each rat. Blood serum was also isolated by centrifugation. Kidneys were removed after perfusion with 0.9% saline solution at 4 °C, followed by decapsulation. The cortex and medulla of the left kidney and the transversely-sectioned half of the right kidney were excised, snap-frozen in liquid N_2_, and finally stored at −80 °C. The other half of the right kidney was fixed in 4% paraformaldehyde and paraffin-embedded for histological studies.

### 4.3. Histological Studies

Hematoxilin/eosin (Sigma-Aldrich, Steinhem, Germany) staining was performed on 5 µm sagittal rat kidney sections. An inverted IX70 microscope (Olympus, Hamburg, Germany) was used for taking microphotographs at 20× and 60× magnification, for histological examination.

### 4.4. Renal Function Indicators

An AutoAnalyzer Cobas 711 (Roche, Basel, Switzerland) was employed for BUN, creatinine, sodium, and potassium determination in serum samples. Creatinine clearance rate was used for GFR calculation. Total protein was determined in urine using the sulfosalicylic acid method [47].

### 4.5. Tissue Homogenization

Kidney cortex and medulla tissues (approximately 50 mg each) were added to 1.5 mL screwcap plastic tubes containing 800 µL of lysis buffer solution: 50 mM Tris–HCl pH 7.5, 125 mM NaCl, 5 mM NaF,1.4 mM Na_4_O_7_P_2_, 1 mM Na_3_VO_4_, and protease inhibitor (Pierce Biotechnology, Inc., Rockford, IL, USA), and 1.5 mm zirconium beads. The tissues were disaggregated on a BeadBug-6 Homogenizer (Benchmark D1036-E, Bechmark Scientific, Sayreville, NY, USA) at 4500 rpm, in three cycles of 90 s. The homogenate was further sonicated for 40 s at 10% of amplitude and centrifuged at 600× *g* for 1 min at 4 °C. An aliquot of the resultant supernatant was diluted 1:10 for total protein determination by the bicinchoninic acid (BCA) protein assay (Pierce Biotechnology Inc., Rockford, IL, USA). The rest of the protein extract was stored at −80 °C until lipidomic analysis.

### 4.6. Lipidomic Analysis by LC-MS/MS

Lipids were extracted from tissues (equivalent to 250 µg of total protein) following Folch’s method [48]. Ten microliters of an internal standard (IS) mix was added before the lipid extraction to obtain the relative molar quantification of lipid species, as previously described [49]. The IS-mixture consisted of the following: CE (16:0-d7), FC-d7, Cer (42:1-d7), HexCer (30:1), LPC (17:0), PC (28:2), PE (32:2), SM (30:1), dhSM (30:0), dhCer (35:0), and Sulf (34:1), in the concentrations given in Table S4. Lipid extracts were dried under a nitrogen stream and reconstituted in 250 µL of acetonitrile/isopropanol (1:1, vol:vol), sonicated for 10 min, and then transferred to an injection vial.

Five microliters of the lipid extract was injected on an LC system Eksigent UltraLC-100 (AB-Sciex LLP, Framingham, MA, USA). The species were separated on a Kinetex C18 column (100 × 2.1 mm, 1.7 µm; Phenomenex, Macclesfield, UK) operating at 55 °C. Elution was applied using a binary mixture of solvent A (60% acetonitrile in water, 10 mM NH_4_COOH) and solvent B (90% isopropyl alcohol in acetonitrile, 10 mM NH_4_COOH) and a linear gradient from 60% A to 100% B in 12 min and 100% B to 60% A in 8 min, at a flow rate of 0.4 mL min^−1^. A QTrap 4000 instrument (AB-Sciex LLP, Framingham, MA, USA) was used for lipid detection, with Analyst 1.6.2 software. Nitrogen was employed both as drying gas (T: 500 °C, pressure: 30 psi) and as nebulization gas (50 psi). The detection was set in electrospray (ESI) positive mode for all lipid classes with the exception of Sulf, which was analyzed in ESI negative mode. CE and FC were analyzed using the atmospheric pressure chemical ionization (APCI) source in the positive ion mode. A targeted approach was used for lipid detection setting multiple reaction monitoring (MRM) transitions for each lipid species at their retention times (Table S5). The LC-MS/MS peak chromatograms were processed using Skyline software version 4.1 (MacCoss Lab, Seattle, WA, USA) [50]. The lipid species were quantified by direct comparison of the area for each species with the area of the IS for their lipid class, as previously described [49]. The results were expressed as nmol/mg protein. Total lipid class levels were determined as the sum of the individual lipid class species quantified. Lipid species were designated according to the recommended notation [51].

### 4.7. Statistical Analysis

Values were expressed as mean ± standard deviation. SPSS 11.5 (SPSS, Chicago, IL, USA) was used for descriptive statistics and evaluating statistical differences in variables between groups by analysis of variance. For two-unpaired-group comparison of lipid variables, either parametric (two-tailed unpaired Student’s t) or non-parametric (Mann–Whitney) tests were performed after normality testing (Shapiro–Wilk). The Benjamini–Hochberg method was used for false discovery rate (FDR) correction of *p*-values when comparing multiple lipid individual species. A two-sided *p*-value < 0.05 and FDR < 0.1 were considered for the identification of statistically significant differences to avoid missing potential biomarker candidates. Fold change of group X versus group Y was calculated as the ratio of the mean values for the variables of the respective groups X:Y.

Multivariate data analysis (MVDA) was performed using SIMCA version 14.1 (MKS Umetrics, Uppsala, Sweden) and MetaboAnalyst 5.0 (https://www.metaboanalyst.ca (accessed on 30 September 2021)) [52], including unsupervised PCA and supervised OPLS-DA. Missing values were substituted using the k-nearest neighbors method. Variables were log-transformed and pareto-scaled prior to MVDA. Models were assessed according to their R^2^ and Q^2^ values. OPLS-DA was validated by permutation test (100 cycles). VIP scores and S-plots from OPLS-DA models were used to identify relevant variables in CISPL versus CNT, CISCIL versus CNT, and CISCIL versus CISPL group discrimination. Simultaneous compliance with VIP > 1, loadings scaled as correlation coefficient |p(corr)ǀ > 0.5, and two-sided *p*-value < 0.05 and FDR < 0.1 for two-group comparison were the criteria employed for potential biomarker selection. AUC values were obtained from the ROC curve plots.

## 5. Patents

The following patents are partially related to the work presented in this manuscript: “Use of cilastatin to reduce nephrotoxicity of various compounds” (patent numbers EP 2,143,429 B1; US 9,216,185 B2; US 9,522,128 B2; and US9,757,349 B2). These are assigned to Fundación para la Investigación Biomédica Hospital Gregorio Marañón (FIBHGM) and licensed by FIBHGM to Telara Pharma S.L. Telara Pharma S.L. has currently entered into a licensing agreement with Arch Biopartners.

## Figures and Tables

**Table 1:** Protective effect of cilastatin during cisplatin-induced renal damage. Serum and urine renal function indicators.

Group	S Cr (mg/dL)	BUN (mg/dL)	GFR (mL/min/100 g)	Prot urine (mg/dL)	FE Na^+^ (%)	FE K^+^ (%)	Vol Urine 24 h (mL)
Control	0.31 ± 0.06	33 ± 7	2.3 ± 0.5	30 ± 6	0.5 ± 0.1	20 ± 5	14 ± 2
Cilastatin	0.33 ± 0.05	30 ± 6	2.0 ± 0.5	28 ± 7	0.53 ± 0.09	21 ± 3	14 ± 6
Cisplatin	1.4 ± 0.8 ^a^	110 ± 70 ^a^	0.4 ± 0.2 ^a^	40 ± 8 ^a^	2 ± 1 ^a^	70 ± 40 ^a^	26 ± 4 ^a^
Cisplatin + cilastatin	0.6 ± 0.2	50 ± 10	1.0 ± 0.3 ^b^	30 ± 5	0.8 ± 0.2	31 ± 9	20 ± 10

^a^ *p* < 0.05 vs. all other groups; ^b^ *p* < 0.05 vs. control group. Abbreviations: SCr, serum creatinine; BUN, blood urea nitrogen; GFR, glomerular filtration rate; Prot urine, proteins in urine; FE, fractional excretion; Vol urine, urinary volume. The results are expressed as mean ± standard deviation (sd) for *n* = 6 animals per group.

**Table 2:** Selected cortex lipids, which are potential biomarkers of cisplatin-induced renal damage. The CISPL group was compared with the CNT group. FC: fold change. *p*-values from univariate analysis are considered. Areas under the curve (AUC) from the receiver operating characteristic curve (ROC) are included. Lipids with *p*-value < 0.05, FDR < 0.1; |p(corr)| > 0.5 and VIP score > 1 from the OPLS-DA model are included.

Lipid (Cortex)	FC	Trend	*p*-Value	p(corr)	VIP Score	AUC ROC
CE 18:0	20.16	↑	0.00079	0.974	3.18	1
CE 18:1	6.62	↑	0.00020	0.983	2.53	1
CE 18:2	5.86	↑	2.28 × 10^−6^	0.964	2.44	1
CE 20:4	8.12	↑	0.00216	0.985	2.64	1
CE 22:6	18.35	↑	0.00088	0.985	3.10	1
Cer 40:1	1.70	↑	0.00074	0.823	1.22	0.944
dhCer 36:0	1.59	↑	0.01193	0.748	1.12	0.917
dhHexCer 34:0	2.00	↑	0.00384	0.721	1.39	0.972
dhHexCer 36:0	1.79	↑	0.01704	0.651	1.27	0.861
dhHexCer 38:0	1.85	↑	0.01409	0.620	1.28	0.861
dhSM 34:0	0.61	↓	0.00866	−0.866	1.23	0.944
dhSM 36:0	1.77	↑	0.00158	0.817	1.24	1
HexCer 34:1	1.50	↑	0.00491	0.737	1.06	0.944
LPC 16:1	0.70	↓	0.00216	−0.903	1.09	1
LPC 18:0	0.67	↓	0.00001	−0.909	1.14	1
LPC 20:3	0.69	↓	0.00018	−0.900	1.08	1
PC 34:5	0.57	↓	0.00010	−0.873	1.33	1
PC 36:5	0.55	↓	0.00016	−0.902	1.37	1
PC 36:6	0.58	↓	0.00001	−0.955	1.32	1
PC 40:6	0.62	↓	0.00433	−0.840	1.18	0.972
PE 36:5	0.54	↓	6.33 × 10^−7^	−0.965	1.44	1
PE 38:3	0.66	↓	0.00782	−0.662	1.07	0.944
SM 34:1	0.71	↓	0.00050	−0.868	1.02	1
Sulf 42:0	0.43	↓	0.00799	−0.870	1.56	1
Sulf OH-40:1	0.36	↓	0.00196	−0.912	1.80	1
Sulf OH-42:1	0.43	↓	0.00219	−0.903	1.59	1
Sulf OH-42:2	0.51	↓	0.00896	−0.799	1.37	0.944

↑: increase induced by CISPL versus CNT. ↓: decrease induced by CISPL versus CNT.

**Table 3:** Selected medulla lipids, potential biomarkers of cisplatin-induced renal damage. CISPL was compared with CNT. *p*-values from univariate analysis are considered. FC and AUCs from the ROC are included. Lipids with a *p*-value < 0.05, FDR < 0.1, |p(corr)| > 0.5, and VIP score > 1 from the OPLS-DA model are included.

Lipid (Medulla)	FC	Trend	*p*-Value	p(corr)	VIP Score	AUC ROC
CE 18:0	18.44	↑	1.98 × 10^−9^	0.969	2.60	1
CE 18:1	5.77	↑	0.0002	0.977	2.01	1
CE 18:2	2.95	↑	0.0001	0.989	1.60	1
CE 20:4	2.88	↑	0.0005	0.942	1.54	1
CE 22:6	5.05	↑	0.0002	0.914	1.82	1
Cer 36:1	1.74	↑	0.0019	0.804	1.13	0.917
Cer 38:1	2.02	↑	0.0003	0.861	1.26	0.972
Cer 40:1	2.52	↑	0.0022	0.928	1.47	1
Cer 42:1	2.26	↑	8.63 × 10^−6^	0.965	1.36	1
Cer 42:2	2.77	↑	0.0022	0.951	1.52	1
dhCer 34:0	1.83	↑	7.71 × 10^−6^	0.948	1.19	1
dhCer 36:0	2.19	↑	0.0001	0.916	1.35	1
dhCer 38:0	2.06	↑	0.0006	0.840	1.27	0.944
dhCer 40:0	1.71	↑	0.0152	0.797	1.09	0.917
dhCer 42:1	1.86	↑	0.0003	0.894	1.17	1
dhHexCer 36:0	2.27	↑	0.0022	0.877	1.35	1
dhHexCer 38:0	1.77	↑	0.0130	0.650	1.16	0.917
dhSM 36:0	1.84	↑	1.23 × 10^−5^	0.933	1.18	1
dhSM 40:0	1.56	↑	0.0071	0.743	1.00	0.944
HexCer 34:1	2.46	↑	1.25 × 10^−6^	0.903	1.42	1
HexCer 36:1	2.47	↑	2.59 × 10^−6^	0.907	1.41	1
HexCer 38:1	1.86	↑	0.0024	0.741	1.19	0.972
PC 34:5	0.39	↓	2.83 × 10^−6^	−0.933	1.46	1
PC 36:5	0.37	↓	0.0022	−0.961	1.55	1
PC 36:6	0.37	↓	0.0001	−0.954	1.55	1
PC 38:7	0.49	↓	0.0001	−0.933	1.28	1
PC 40:7	0.51	↓	0.0022	−0.953	1.26	1
PE 34:1	0.61	↓	0.0022	−0.881	1.08	1
PE 34:2	0.59	↓	0.0001	−0.933	1.10	1
PE 34:3	0.49	↓	1.28 × 10^−5^	−0.973	1.29	1
PE 36:4	0.51	↓	0.0022	−0.951	1.27	1
PE 36:5	0.26	↓	0.0022	−0.989	1.77	1
PE 38:3	0.64	↓	0.0002	−0.922	1.02	1
PE 38:4	0.61	↓	0.0005	−0.889	1.05	0.972
PE 38:5	0.48	↓	0.0022	−0.969	1.31	1
PE 38:6	0.51	↓	0.0022	−0.920	1.26	1
PE 38:7	0.45	↓	0.0001	−0.955	1.37	1
PE 40:4	1.83	↑	0.0043	0.841	1.16	0.972
SM 34:1	0.61	↓	0.0022	−0.872	1.07	1
Sulf 40:2	0.40	↓	0.0022	−0.777	1.48	1

↑: increase induced by CISPL versus CNT. ↓: decrease induced by CISPL versus CNT.

**Table 4:** Selected cortex lipids that are potential biomarkers of cilastatin nephroprotection. The CISCIL group was compared with the CISPL group. *p*-values from univariate analysis are considered. FC and AUC from the ROC are included. Lipids with *p*-value < 0.05, FDR < 0.1, |p(corr)| > 0.5, and VIP score > 1 from the OPLS-DA model are included.

Lipid (Cortex)	FC CISCIL vs. CISPL	FC CISPL vs. CNT	FC CISCIL vs. CNT	*p* Value CISCIL vs. CISPL	p(corr)	VIP Score	AUC ROC
CE 18:0	0.40 ^a^	20.16 ^a^	8.08 ^a^	0.0051	−0.912	2.49	1
CE 18:1	0.44 ^a^	6.62 ^a^	2.94 ^a^	0.0004	−0.912	2.41	1
CE 18:2	0.38 ^a^	5.86 ^a^	2.24 ^a^	0.0004	−0.896	2.57	1
CE 20:4	0.40 ^a^	8.12 ^a^	3.25 ^a^	0.0007	−0.931	2.53	1
CE 22:6	0.32 ^a^	18.35 ^a^	5.83 ^a^	0.0031	−0.935	2.80	1
Cer 40:1	0.71 ^a^	1.70 ^a^	1.20 ^a^	0.0060	−0.714	1.44	0.889
dhHexCer 34:0	0.57 ^a^	2.00 ^a^	1.14	0.0117	−0.709	1.88	0.917
PC 32:2	0.80 ^a^	1.12	0.89	0.0053	−0.787	1.21	0.944
PE 36:5	1.21 ^a^	0.54 ^a^	0.65 ^a^	0.0118	0.746	1.08	0.889
Sulf 38:1	0.78 ^a^	1.16	0.90	0.0101	−0.758	1.31	0.861
Sulf 40:2	0.40 ^a^	1.00	0.41 ^a^	0.0028	−0.797	2.17	1

^a^ *p*-value < 0.05 with FDR < 0.1.

**Table 5:** Selected medulla lipids that are potential biomarkers of cilastatin nephroprotection. The CISCIL group was compared with the CISPL group. *p*-values from univariate analysis are considered. FC and AUC from the ROC are included. Lipids with a *p*-value < 0.05, FDR < 0.1, |p(corr)| > 0.5, and VIP score > 1 from the OPLS-DA model are included.

Lipid (Medulla)	FC CISCIL vs. CISPL	FC CISPL vs. CNT	FC CISCIL vs. CNT	*p*-Value CISCIL vs. CISPL	p(corr)	VIP Score	AUC ROC
CE 18:2	0.64 ^a^	2.95 ^a^	1.89 ^a^	0.0039	−0.864	1.53	0.917
Cer 42:2	0.69 ^a^	2.77 ^a^	1.92 ^a^	0.0087	−0.720	1.36	0.944
dhHexCer 36:0	0.62 ^a^	2.27 ^a^	1.40	0.0022	−0.637	1.45	1
HexCer 34:1	0.72 ^a^	2.46 ^a^	1.78 ^a^	0.0123	−0.659	1.26	0.972
PC 32:1	1.32 ^a^	0.63 ^a^	0.84	0.0047	0.741	1.04	0.944
PC 36:5	1.58 ^a^	0.37 ^a^	0.58 ^a^	0.0087	0.817	1.44	0.944
PC 36:6	1.71 ^a^	0.37 ^a^	0.63 ^a^	0.0030	0.875	1.63	0.944
PE 34:2	1.51 ^a^	0.59 ^a^	0.89	0.0082	0.689	1.24	0.944
PE 34:3	1.63 ^a^	0.49 ^a^	0.80	0.0022	0.935	1.54	1
PE 36:3	1.29 ^a^	0.76 ^a^	0.98	0.0040	0.794	1.04	0.944
PE 36:4	1.57 ^a^	0.51 ^a^	0.80	0.0087	0.654	1.29	0.944
PE 36:5	1.94 ^a^	0.26 ^a^	0.51 ^a^	0.0022	0.960	1.78	1
PE 38:5	1.55 ^a^	0.48 ^a^	0.74 ^a^	0.0022	0.854	1.39	1
PE 38:6	1.54 ^a^	0.51 ^a^	0.79	0.0130	0.811	1.44	0.917
SM 42:1	1.49 ^a^	0.71 ^a^	1.07	0.0115	0.756	1.33	0.889
Sulf 40:2	2.47 ^a^	0.40 ^a^	0.98	0.0049	0.720	2.01	0.917

^a^ *p*-value < 0.05 with FDR < 0.1.

## Data Availability

The data presented in this study are available in Supplementary Materials: Table S1.

## Supplementary Materials

The following are available online at https://www.mdpi.com/article/10.3390/ijms222212521/s1, Table S1: LC-MS/MS lipidomics analysis of cortex and medulla lipids in rats treated with cisplatin and/or cilastatin. Table S2: Statistical analysis of kidney cortex lipid species. Table S3: Statistical analysis of kidney medulla lipid species. Table S4: Concentration of lipid species in the internal standard mix used on experiments. Table S5: List of MRM transitions, retention time, and mass spectrometer settings used in the study.
